# Prototype System for Measuring and Analyzing Movements of the Upper Limb for the Detection of Occupational Hazards

**DOI:** 10.3390/s20174993

**Published:** 2020-09-03

**Authors:** Dolores Parras-Burgos, Alfonso Gea-Martínez, Lucas Roca-Nieto, Daniel G. Fernández-Pacheco, Francisco J. F. Cañavate

**Affiliations:** Department of Structures, Construction and Graphic Expression, Universidad Politécnica de Cartagena, 30202 Cartagena, Spain; alfonsogea.m@gmail.com (A.G.-M.); lucas.roca@upct.es (L.R.-N.); daniel.garcia@upct.es (D.G.F.-P.); francisco.canavate@upct.es (F.J.F.C.)

**Keywords:** low-cost tool, musculoskeletal disorder, risk factors, ergonomics, 3D modelling

## Abstract

In the work environment, there are usually different pathologies that are related to Repetitive Efforts and Movements (REM) that tend to predominantly affect the upper limbs. To determine whether a worker is at risk of suffering some type of pathology, observation techniques are usually used by qualified technical personnel. In order to define from quantitative data if there is a risk of suffering a pathology due to movements and repetitive efforts in the upper limb, a prototype of a movement measurement system has been designed and manufactured. This system interferes minimally with the activity studied, maintaining a reduced cost of manufacture and use. The system allows the study of the movements made by the subject in the work environment by determining the origin of the Musculoskeletal Disorder (MSD) from the movements of the elbow and wrist, collecting data on the position and accelerations of the arm, forearm and hand, and taking into account the risk factors established for suffering from an MSD: high repetition of movements, the use of a high force in a repetitive manner, or the adoption of forced positions. The data obtained with this system can be analyzed by qualified personnel from tables, graphs, and 3D animations at the time of execution, or stored for later analysis.

## 1. Introduction

In the workplace, workers are often unaware of the relationship between the discomfort they experience on a daily basis and the repetitive tasks or efforts they undertake in their jobs. In this sense, the different pathologies that can be related to Repetitive Efforts and Movements (REM) usually predominantly affect the upper limbs [1,2,3]. Currently, the methods for assessing whether workers are at risk of suffering a pathology due to repetitive movements and efforts in the upper limb are based on observation techniques by qualified technical personnel, although there are studies that implement integrated sensors for smart phones to discreetly monitor workers’ body postures, autonomously identifying possible ergonomic risks related to the work [4]. These studies are focused on trunk and shoulder flexions, and are considered very close to measurements by observation.

A risk factor assessment is necessary to determine if there is a possibility of a Musculoskeletal Disorder (MSD) due to the job. This assessment will depend on the type of work performed and its associated risks. The International Ergonomics Association (IEA) and the ISO Technical Committee on Musculoskeletal Disorders have defined a general model, validated by the International Commission on Occupational Health (ICOH), of the main risk factors that should be considered, presenting observation procedures that can be used in their description, classification and evaluation [5,6,7,8]. Among the most common actions in the workplace are: repetitive work, manual handling of loads, forced postures, and application of force [9,10,11,12]. This study focuses on evaluation methods for repetitive work and forced postures, since this is where a movement capture system can have the greatest influence:

i. Repetitive movements: The OCRA method (OCcupational Repetitive Action) is the method proposed for a detailed evaluation of work where repetitive movements of the upper limb exist, associating the level of risk with the predictability of the appearance of a disorder in a given time [8,9,10]. This method of evaluation considers different factors such as recovery periods, frequency of actions, use of force, presence of uncomfortable postures, and additional factors such as vibrations or inadequate equipment. The OCRA method has been established by international consensus as the preferred method for assessing the risk of repetitive work in the upper limb in ISO 11228-3 and UNE-EN 1005-5. With this method, a value called the OCRA index is obtained that will indicate if there is a high risk, a very low risk, or if there is no risk of suffering an MSD. In addition to the OCRA method, there are different risk assessment methods for work with repetitive movements: the Strain index, the Keyserling Checklist, the Risk assessment method for repetitive movements proposed by the PMVS (Specific Health Surveillance Protocols), and the Risk assessment method for pressure neuropathies proposed by the PMVS [13,14,15,16]. In these named assessment methods, the assessment of the angles formed in the joints is done by direct observation or by video analysis, and therefore a more objective system of assessment is needed.
ii. Forced postures: The use of ISO 11226:2000 is proposed for the evaluation of risks associated with work in forced postures [17]. This standard considers the angles in the joints and the time that they are maintained in static postures where force is not applied or is minimal. Several areas of the body are considered independently, such as the trunk, head and neck, and upper limbs. In a first step to determine if a posture is “Acceptable”, if there is a need to go to the next step, or if it is directly “Not recommended”, the joint angles and the existence of supports must be considered. In a second step, the time that the evaluated posture is maintained is analyzed to obtain as a result if the posture is “Acceptable” or “Not recommended”.

The choice of an appropriate method of analysis to determine possible pathologies depends on the nature of the research and the purpose for which the data will be used. Some methods are suitable for use only by expert researchers and require a large number of resources, and other techniques allow for observational assessments and are more focused on occupational safety and health professionals, who have limited time and fewer resources at their disposal [14]. Observation-based methods range from checklists and diagrams using software [15,16,17], questionnaires for quantitative ergonomic analysis [18], to video observation or direct measurement [18]. Some of these methods, such as those based on observational measurements of posture from videos, have been studied and offer great confidence in the results obtained [19]. Other studies propose methods that analyze the articulated posture, which predicts the angles of the body’s joints from a single depth image [19], or that take quick snapshots with a smartphone to recognize angles, thus analyzing users’ positions [20]. In this line, but with a system of pressure sensors, a classification of human standing posture from standing-pressure images is proposed, by means of which a robot system can predict the foreseen actions of human workers in a HRC environment (Human Robot Collaboration) [21]. Other promising studies use sensors to detect joint position without connecting them directly to the study subject, but further study is needed for its use in real-life settings: the use of Kinect™ range sensors in observational methods for assessing postural loads [22], the recognition of postural patterns through wearable sensors and machine-learning algorithms fed with kinematic data [23], or a multi-parametric wearable system based on two fiber Bragg grating sensors for monitoring neck movements and the breathing activity of computer workers [24].

According to the literature, the most commonly used methods for analyzing the upper limb are based on direct observation of the worker by qualified technical personnel, obtaining values for the angles formed by the joints and the time each one is maintained in an estimated and subjective manner [25,26]. For this reason, it is necessary to use technologies and instrumentation that interfere as little as possible with the worker, that are light and portable, and that allow the collection and storage of more objective measurements in an automatic way [25,27,28].

In order to determine the relationship between the pathology and the working environment of the workers in a more objective way, a system has been designed that is capable of evaluating the movements of one of the upper limbs based on quantitative data. This study has focused on measuring the accelerations and postures adopted by the upper limb and showing this data in a graphical timeline. This will allow technical personnel to have objective data and improve the accuracy and reliability of diagnoses. Taking into account that there is a higher percentage of right-handed people (85–90%) [29,30], it has been considered to start the investigation by analyzing the right upper limb. The study focuses on elbow and wrist movements, collecting data on arm, forearm, and hand position and accelerations, and taking into account the risk factors for MSD listed by the World Health Organization (WHO) [31,32]: high repetition of movements, repetitive use of high force, and forced positions. In determining whether a position is forced, the nature of the joints involved should be considered.

## 2. Materials and Methods

In order to determine the relationship between the pathology and the work environment, a prototype was designed and manufactured that is capable of measuring and storing, for later analysis, the movements made by a subject’s right upper limb. The system consisted of three interconnected parts: a mechanical part in the form of an exoskeleton, an electronic part or control system, and a portable device with an Android OS software application.

### 2.1. Electronic or Control Part

The electronic or control part of the motion capture system was responsible for taking the data of the movements made and storing it for analysis. It was also in charge of establishing a Bluetooth connection with a portable device through a software application in Android OS. The main components of this part are shown below:

(a) Microcontroller: Arduino Mega 2560 rev3 board. This board has more computing power than the rest of the 8-bit Arduino boards, as well as a larger number of analog and digital inputs and outputs (54 digital inputs/outputs and 16 analog inputs/outputs). To communicate with all the components used:
  i. Serial port communication: Arduino Mega and Arduino Due. They have four UART (Universal Asynchronous Receiver-Transmitter) TTL 0V/5V units. One unit was used to connect with a computer, and another one for the connection via Bluetooth with the mobile device. In both applications a speed of 9600 bauds was used.
  ii. I2C (inter-integrated circuit) communication: Three 3-axis accelerometers with high resolution (13-bit) measurement (model ADXL345) and a 16 × 2 LCD (Liquid Crystal Display). Each of the components has a specific i2c address. The Arduino board worked as a master, setting the clock speed, and requesting the data from each component separately through its i2c address.
(b) Accelerometers: Three 3-axis accelerometers with high resolution (13-bit) measurement (model ADXL345). A SparkFun Bi-Directional Logic Level Converter was used, to be able to connect and read the data correctly from the three accelerometers, because these accelerometers have two i2c directions.
(c) Display: 16 × 2 character LCD display and i2c connection for easy operation of the measuring system. This display, together with the buttons next to it, permitted us to navigate through the various menus, as well as to activate and deactivate data collection.
(d) SD card reader: SD card reader connected to Arduino Mega board through SPI (Serial Peripheral Interface) communication.
(e) Shield: ProtoShield. This shield, with dimensions 106 × 56 mm, allowed us to weld almost any available component to it. It consisted of a Perfboard-like grid where the resistors, connectors, voltage adapter, Bluetooth, and microSD reader have been soldered.
(f) Bluetooth: Bluetooth HC-05 module connected through one of the four serial ports of the Arduino Mega board, with a speed of 9600 baud.
(g) Potentiometers: Three rotary potentiometers type B of 10 k with linear variation and a maximum turn of 300° and a rotary “multiturn” potentiometer of 10 k with linear variation and a turn of 3600° and 10 turns. While the first three do not reach a full turn, the multiturn potentiometer is used to turn the forearm, which is transmitted to the potentiometer through a gear.

**Figure 1:**
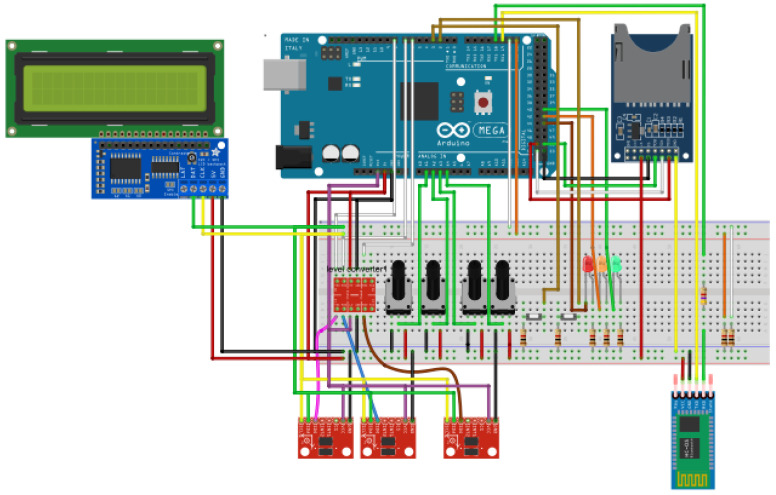
Design of the electronic or control system implemented in the Fritzing software.

**Figure 2:**
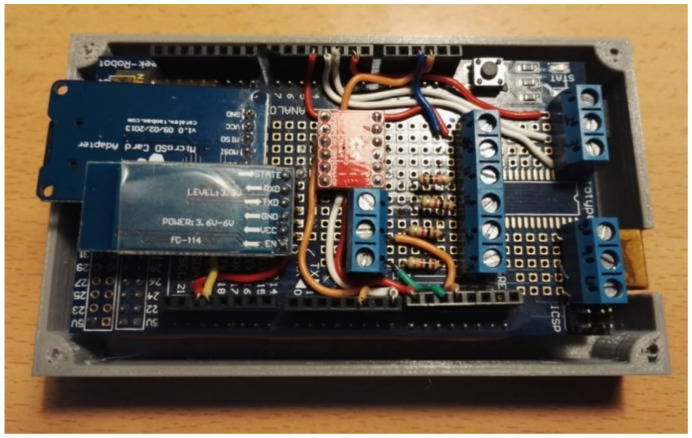
Mounting on the shield.

The electronic system was designed using the Fritzing software (open-source software for electronic design automation, Interaction Design Lab), in which each of the elements that make up the system were integrated (Figure 1). In a first stage of implementation, all the elements were mounted on a Protoboard to check the correct operation of the system. Later, the system was implemented on the shield by mounting the microSD card reader, the Bluetooth module, a voltage adapter for the accelerometers, and several connectors to connect the other elements that will be outside the box: two control buttons, LCD screen, potentiometers, and accelerometers (Figure 2).

**Figure 3:**
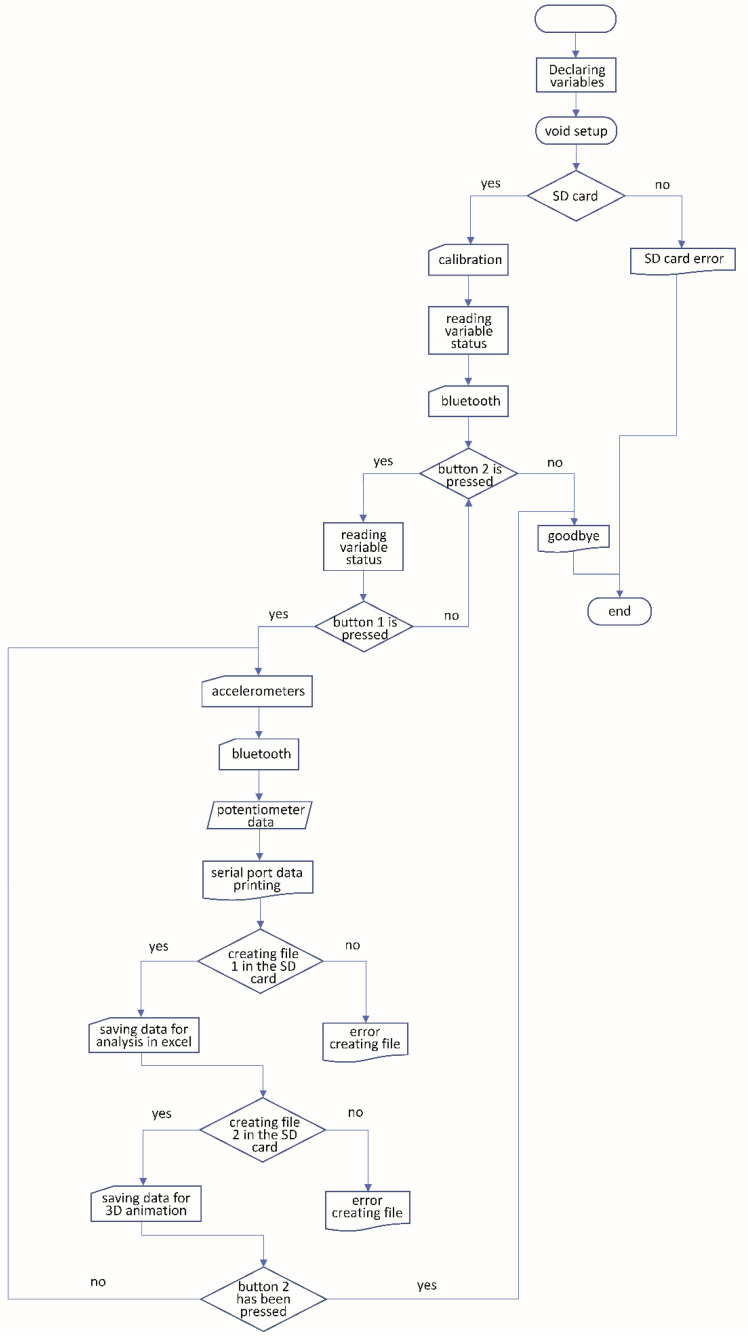
Flow chart of the code developed for Arduino.

A brief description of the flowchart of the electronic system with all the communications between the elements would be the following (Figure 3):

1. When the system starts, it checks the existence of an SD card inside and whether it is writable or not. If this is not possible, it will show an error on the screen and will not allow the test to continue.
2. If the card check is correct, the system will be calibrated by keeping the exoskeleton in a straight position. Once the calibration has been performed, the device will show on the screen the possibility to start the test by pressing button 1, or to end it before starting by pressing button 2.
3. When the test starts, the data from the accelerometers, the Bluetooth module, and the potentiometers will be read and stored in two different files inside the SD card: one file to export the data to an Excel spreadsheet, and another one for the 3D animation. In the event of an error when writing to the SD card, an error will be indicated on the LCD screen and the test will be completed. This will be done in a cyclic manner until button 2 is pressed and the test is finished.

### 2.2. Exoskeleton

The exoskeleton, or mechanical part of the capture system, is in charge of supporting the electronic part and transmitting the arm movements to the different sensors. This aspect differentiates it from most of the exoskeletons in the market, which are made to facilitate movements of people with reduced mobility, or to facilitate actions in which heavy loads are lifted [33,34,35]. One such movement capture system is PrioVR, and more specifically the Yost Labs 3-Space Micro USB module (https://yostlabs.com/product/3-space-micro-usb/), which only integrates accelerometers and gyroscopes as sensors. For this reason, and to offer more reliability and accuracy when calculating the angles formed in the joints, the system proposed in this work also integrates a potentiometer into the exoskeleton for each of the joint axes from the elbow to the wrist.

**Figure 4:**
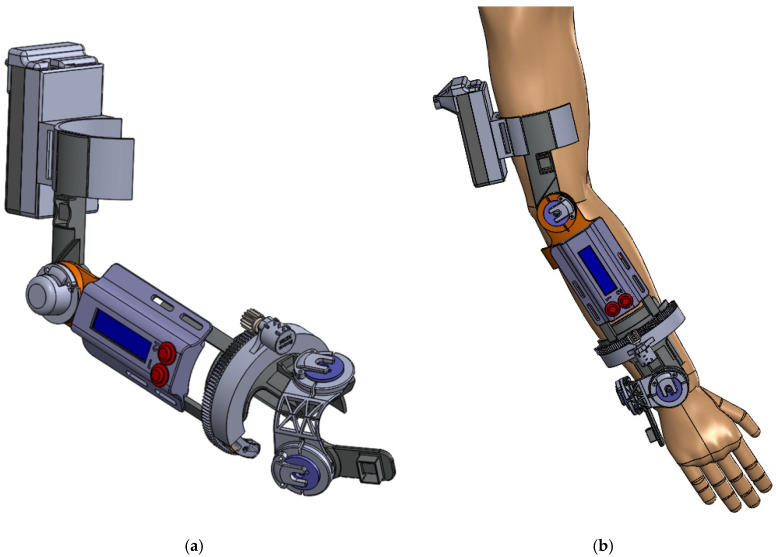
(**a**) 3D Modelling of the final prototype assembly, (**b**) Prototype mounted on the arm of a subject.

The motion capture system was designed using SolidWorks design software (Dassault Systemes, Vélizy-Villacoublay, France). The initial design was based on an anatomical model with a 95th percentile, ensuring that with slight adjustments, the motion capture system can be adapted to the largest possible number of subjects. The followed modelling method consisted of designing each of the pieces separately, always taking into account the maximum size and the limitations of the chosen manufacturing system to make the prototype. Once all the components had been designed, different subassemblies were created, thus defining the functional assemblies of the different fixed parts and leaving the possibility of movement in the joints presented by the prototype. The system consisted of 29 different parts whose final assembly is shown in Figure 4.

**Figure 5:**
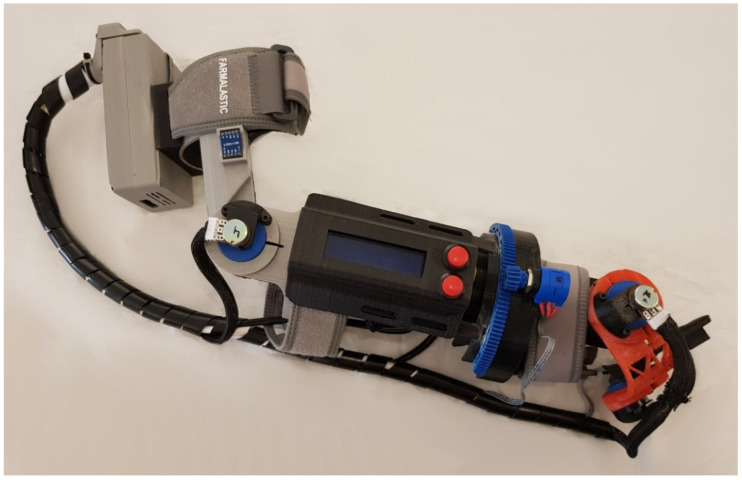
System prototype.

For the construction of the prototype, different manufacturing processes were considered, finally selecting the additive manufacturing method by means of molten plastic deposition or FDM (Fused Deposition Modeling). This method has several advantages such as the speed and low price required to create a functional prototype, in addition to being able to make really complex shapes. A Prusa i3 printer from the company RepParts3d was used to manufacture this prototype, capable of working with thermoplastics with a melting point of less than 250° and equipped with a heatable printing surface or “hot bed”. This printer has a working volume of 180 × 200 × 180 mm. A large number of parts of the prototype were manufactured with PLA (PolyLactic Acid), while the parts that suffer wear, such as the bushes and gears of the potentiometer used for the longitudinal rotation of the forearm, were designed to be manufactured with Iglidur^®^ I150-PF. Once all the components were manufactured, the electronic part was incorporated into the system. In Figure 5, the final prototype is shown, with the assembly of all the parts of the system. The total weight of the prototype is 885 g, and being so, is considered quite a light model.

The box in which the electronic or control part is mounted was also made using 3D printing, just as the rest of the exoskeleton. The design took into account the necessary cooling of the Arduino, creating a box as open as possible, thus leaving part of the electronic part visible.

### 2.3. Portable Control Device

The gesture control of the hands was done by direct observation of a qualified person. To facilitate data collection, an application was developed for tablets with Android OS, in which the observer can start and stop the data collection from the measurement system, as well as select which type of grip the worker under observation is performing. In order to avoid the physical link between the observer and the worker, Bluetooth communication between the measurement device and the tablet has been chosen. This technology allows a sufficient observation distance so as not to interfere with the movements of the worker. The application was developed using the Android Studio IDE.

**Figure 6:**
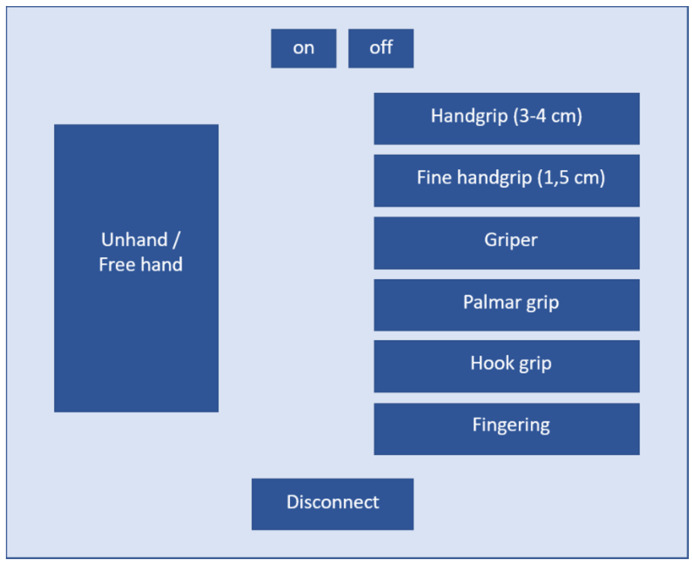
Screenshot of the application.

The application was designed to be user-friendly and intuitive. The first screen is used to pair the measuring device with the tablet. Once the two devices are paired up, the screen is accessed where data collection is activated, and the types of grip the worker is performing are recorded. Figure 6 shows the screen that has been designed with only the right-hand data recording in mind.

This application has been programmed to send text strings via Bluetooth to the measuring device. This device stores these strings along with the position and acceleration data, creating a database that allows the workers’ movements to be studied and recreated. The types of grip that the observer marks in the Android application are also displayed on the LCD screen of the measuring device as a check that everything is working correctly.

### 2.4. Sample Data

The system developed provides two ways of displaying all the data being collected by the measuring devices. One of them is by means of a 3D animation, in which the user can see the movements captured at the same moment of the data capture or see it later to be able to analyze the movements. The other form of data visualization is a spreadsheet file, where the movements, accelerations, and types of grip performed by the worker are displayed graphically, and from which a summary of the movements performed by the subject is obtained.

**Figure 7:**
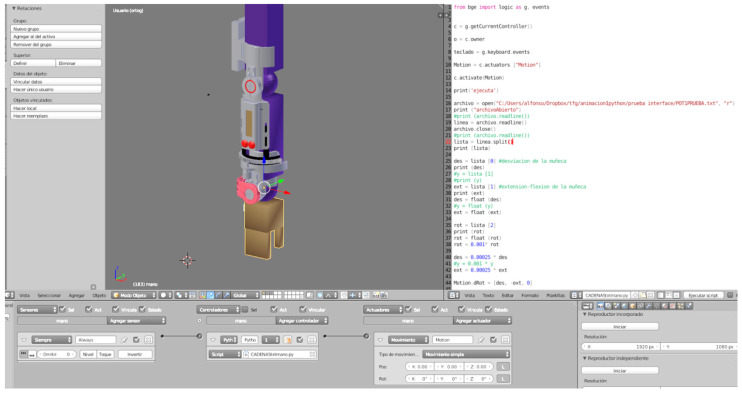
System modeling in Blender.

In relation to the 3D animation, the animation and modeling software Blender (free and open source 3D creation suite, Blender Foundation) was used to visualize the movements made by the worker from different angles of vision (Figure 7). Different scripts in Python and the “Game Logic” system integrated in Blender have been used to give movement to each one of the joints (wrist, elbow, and forearm rotation in its longitudinal axis), creating the appearance of an arm that performs the same movements captured in the worker. The visualization can be done during the test, or once the data has been entered into the computer, allowing a more detailed analysis of the data obtained.

**Figure 8:**
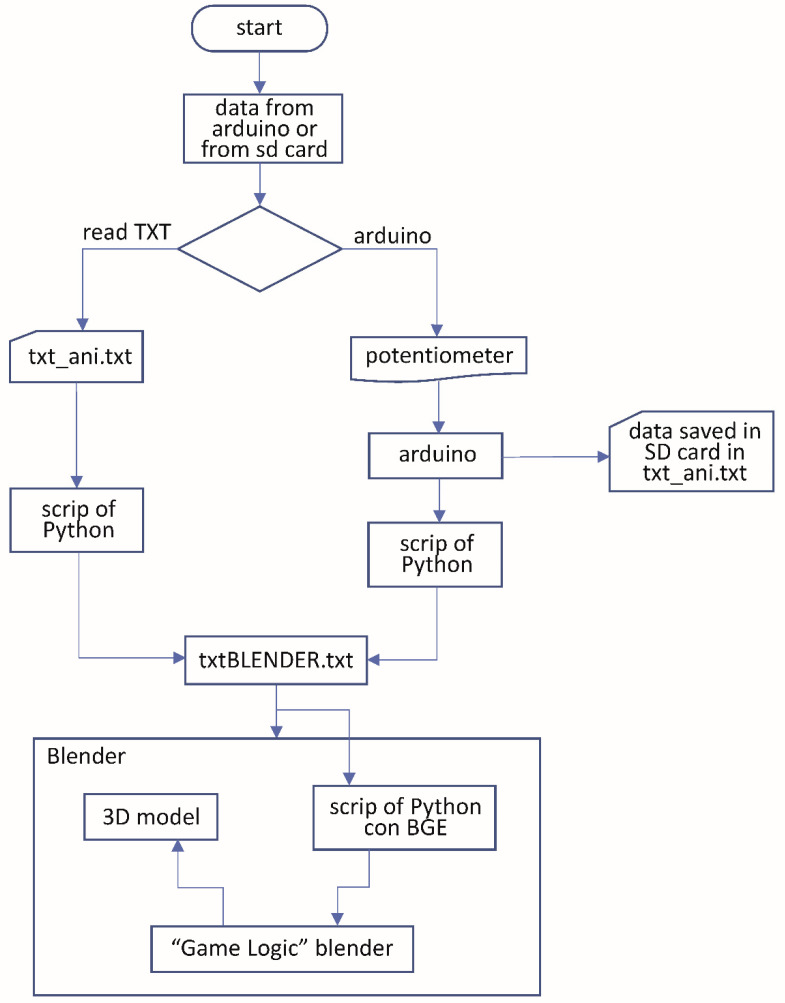
3D animation data flowchart.

If the movements are to be visualized at the same time as they are performed by the subject, the measuring device must be connected to the PC by means of a USB cable, and the communication between the two devices is carried out through the serial port. The communication between the sensors and the 3D animation is made following the diagram shown in Figure 8. Once the measurement device was connected or the SD card inserted in the reader, a software created in the Python programming language version 2.7 was executed by means of two small scripts that allowed us to read and export the data to Blender. With this script, a window is created with two buttons that will offer the option to choose the origin of the data that we want to show in the 3D animation. The user can choose between the Arduino serial port showing on the screen the movements that the worker is doing at that moment, or data can be read from a TXT file that will have been copied to a predetermined location, thus being able to show in the 3D animation the movements that were previously registered in the SD card of the measuring device.

Taking into account the described process, a movement logic has been created for each arm joint that refers to a Python script. These movement logics have been created for the wrist, elbow, and forearm rotation movement on its longitudinal axis. Each of them calls a different Python script. With this script, positions one and two of an alphanumeric string can be read from the TXT file. For the other movements, different positions of this same string are read. In Table 1 is shown a row of this file, where the relative values of the sensors of the arm with respect to the previous instant can be seen. The numbers marked in red colour correspond to the values of the potentiometers, while those marked in black correspond to the raw values of the accelerometers.

## 3. Results

A test with the designed prototype was carried out in a laboratory with a healthy volunteer with no previous ailments, to evaluate the movements executed and to extrapolate them to any worker performing the same activity. The data and graphs obtained in this test are shown in this section as an example.

**Figure 9:**
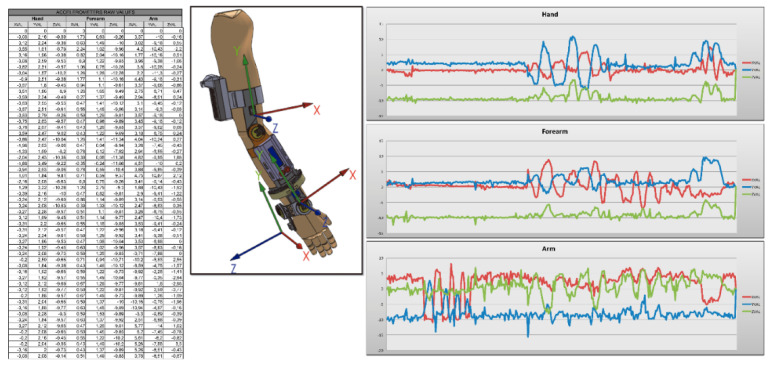
Data collected by accelerometers.

**Figure 10:**
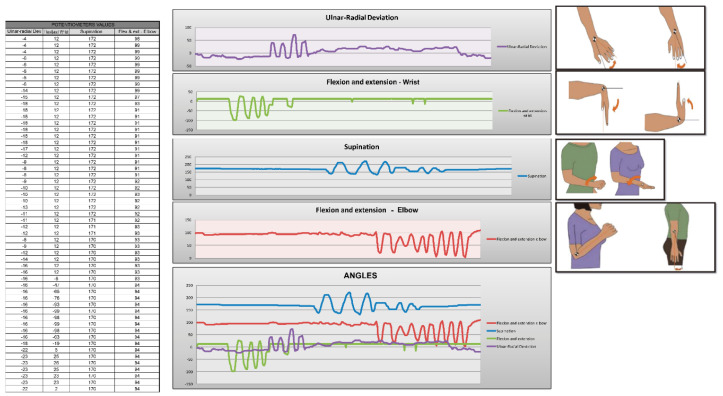
Data collected by the potentiometers.

**Figure 11:**
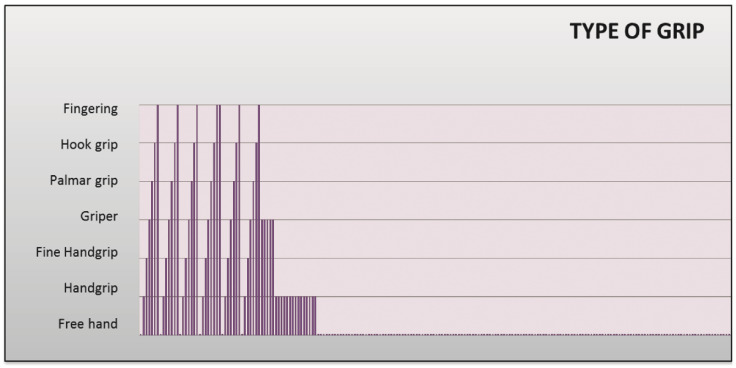
Graph of the types of grip.

An Excel spreadsheet template has been created for the display and subsequent analysis of the data obtained. Due to the large amount of data obtained, these can be difficult to interpret by a person not used to it, so to facilitate the analysis process, different formulas and graphs have been implemented. The data obtained from each sensor is separated according to its origin: the user can see the data obtained by the accelerometers (Figure 9), those obtained by the potentiometers (Figure 10), and by the observer in the Android application (Figure 11). Besides being able to display different graphs with the data, a button has been implemented that allows a quick analysis of the data and obtaining a report in PDF format.

**Figure 12:**
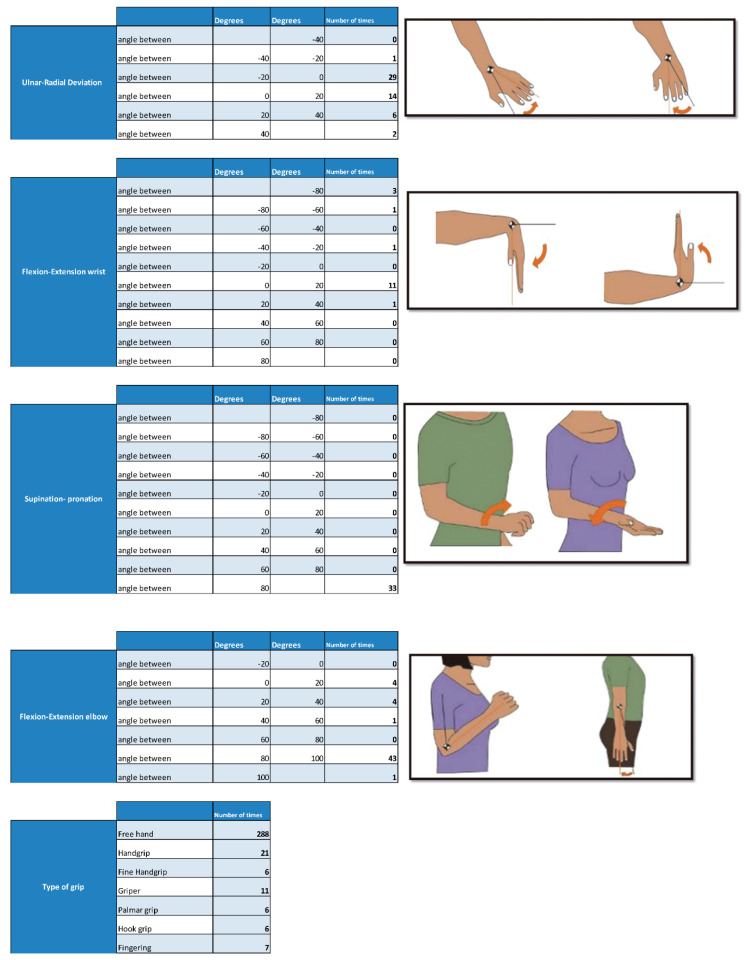
Analysis of angles formed in the wrist, elbow and types of grip.

To analyze the data, the times that a certain value has been a relative maximum or minimum in the different graphs are counted (Figure 12). The report obtained in PDF shows the angles obtained with the potentiometers observing the accelerations that have taken place and determining in this way if they have been higher than certain values. These values or ranges can be easily modified by editing the tables, which allows for more detailed or personalized studies to be carried out on each subject. The report also indicates the actions that were being performed with the hand at each moment, as well as the number of times each of these actions has been performed.

**Figure 13:**
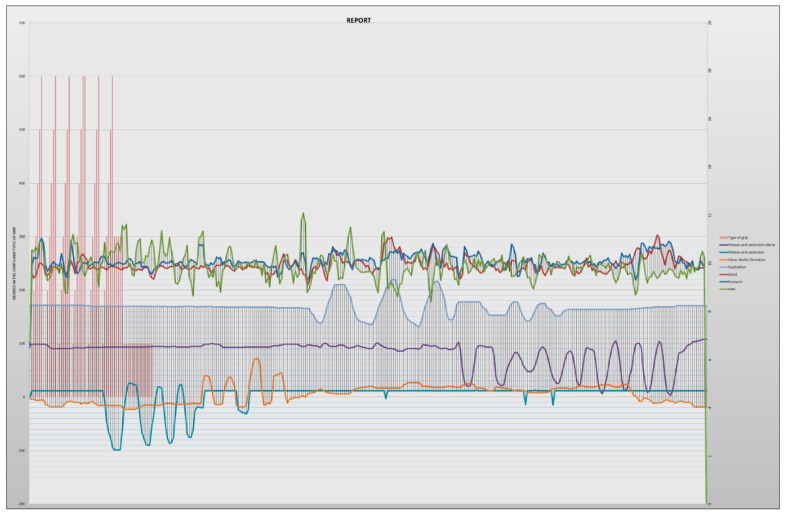
Graph obtained from the report.

In the final part of the report, a graph is shown together with all the data taken by the motion capture system and the observer. The purpose of this table is to be able to relate the angles taken in the joints with the accelerations suffered and the type of grip, which, when shown in the same time line, is easily relatable (Figure 13).

## 4. Discussion

The objective of this work was to develop a lightweight and portable technology that would interfere as little as possible with the user. According to some other studies analysed (Table 2), most of the existing works use sensors attached to different parts of the body that serve to collect data and information on different user movements. The prototype presented in this work requires an exoskeleton (due to the potentiometers attached to the joint axes) that has been designed to adapt to the user with a minimum weight. Even so, it is believed to improve the interference that this system can produce with the user by optimizing its handling. The main advantages of the developed technology are: (i) the union of potentiometers and accelerometers to capture the movements, which are considered more precise, and (ii) the graphs and tables with objective data offered by the system for evaluation by qualified technical personnel for the detection of musculoskeletal disorders.

In addition, the tests carried out in this study confirmed that the proposed measurement system can capture the movements made by the workers and detect those that may be harmful to their health. In this way, the results obtained in the tests can allow a redesign of the workplaces or a change in the tasks that prevent occupational risks. On the other hand, and taking into account the tests carried out in the laboratory, the following improvements are proposed in future versions of the prototype:

(a) The prototype should be better adjusted so that the worker can perform the movements naturally and without difficulty. Perhaps customized fastenings of the system could be manufactured according to each user, by means of 3D printing.
(b) The accelerometer data in the 3D animation can be improved to give more quality and realism.
(c) Expand user control zones by adding shoulder movements.
(d) Use the accelerometers as vibrometers. Many occupational diseases are caused by vibrations, being named as a risk factor in some evaluation methods, but they are not measured unless they are one of the main causes of the disease.
(e) Offer a new way of analyzing data using templates similar to the OCRA method to show by means of a scale the level of risk of a worker to suffer an MSD.

## 5. Conclusions

The present study offers an objective method to detect the different actions that suppose some risk of suffering an MSD in the labor scope, measuring the accelerations suffered in the right superior extremity and the adopted postures. All the data obtained are offered in a table relating these postures and the accelerations against time, making it possible to determine the frequency of a task and the times that elapse between the movements performed. For the application of this new method, a prototype has been designed and built by means of 3D printing with the FDM method, using different materials with printing configurations adapted to each of them. The total printing time has been estimated at 150 h. A system of sensors connected to an Arduino board has also been developed to measure and store the movements performed. In addition, the system allows for the generation of a table capable of summarizing and graphically displaying the data collected for subsequent analysis by qualified technical personnel. In order to facilitate the reading of the data obtained, the system shows a 3D animation that visualizes the movements made in the elbow and wrist joints at the same time of the execution or later.

As future lines of research, we will seek to improve the ergonomics of the measurement system by adapting it to each subject, by means of a structure manufactured with 3D printing from the 3D model of the scanned study arm. On the other hand, the aim is to improve the data from the accelerometers in the 3D animation to give it more realism and add movement from the shoulder, as well as to use the accelerometers as vibrometers. Another study improvement would be to directly analyze the data obtained in a template similar to the OCRA method to show by means of a scale the level of risk of a worker to suffer an MSD.

## Figures and Tables

**Table 1:** Relative values of the sensors.

0	0	0	0	0.00	0.82	−9.89	1.53	1.29	−9.92	2.51	−9.30	−1.53
0	0	0	−30	−1.06	0.31	−9.38	0.12	1.96	−9.77	2.00	−10.16	0.39
0	0	0	−41	−0.47	2.00	−10.00	1.96	1.14	−10.36	2.24	−10.75	−2.31

**Table 2:** Examples of MSD measurement technologies.

Authors	Systems and Sensors	Description
[4]	Smartphone sensors (Accelerometer, Gyroscope and Linear acceleration)	A smartphone held on the arm and another one on the user’s waist to perform the study.
[36]	Smartphone sensors (Accelerometer and Gyroscope)	A smartphone held on the user’s arm.
[37]	External Musculoskeletal Joint Angle sensor system (Magneto-resistive angle sensor)	The sensing element and the magnet are tightly mounted on the torso and the upper arm, respectively. The rest of the sensor system is enclosed in 12 × 8 × 6 cm lightweight steel box. This box is to be mounted on the worker’s belt.
[21]	Standing Postures Classification System (SPCS) (A pressure-sensing floor)	A human worker standing on the floor generates a foot-pressure distribution over the sensor matrix, which is converted to a greyscale image by the data acquisition system of the SPCS.
[22]	Kinect™ range sensor (Depth sensor)	Range sensors can detect the position of the joints at high sampling rates without attaching sensors or markers directly to the subject under study.
[23]	Wireless inertial measurement units (IMUs)	Eight wireless inertial measurement units are used to gather kinematic data of the upper and lower body segments of each subject. To limit relative movements between each sensor and its body segment, each IMU is fixed with elastic straps.
[24]	The fiber Bragg grating (FBG)-based flexible sensor.	Sensors positioning is evaluated to ensure high sensor capability in detection and discrimination of different neck movements and breathing activity. A polyacrylate bandage is used to allow a better adhesion and compliance to the skin. This bandage features adhesiveness, elasticity, and high breathability.
[38]	The bidirectional long short-term memory (Bi-LSTM), a deep learning architecture (Wearable inertial measurement unit, WIMU)	Using a wearable inertial measurement sensor to monitor a worker’s bodily movements, this study investigates the feasibility of identifying various physical loading conditions by analyzing a worker’s lower body movements.
[39]	Wearable sensing technology combined with predictive modeling (3-D accelerometer, gyroscope, and magnetometer sensor)	Four commercial inertial sensors are attached on the participant at the sixth thoracic vertebra (T6), the first sacral vertebra (S1), and superior aspect of the right and left shank midway between the lateral femoral and malleolar epicondyles.
